# Survival Online: a web-based service for the analysis of correlations between gene expression and clinical and follow-up data

**DOI:** 10.1186/1471-2105-10-S12-S10

**Published:** 2009-10-15

**Authors:** Luca Corradi, Valentina Mirisola, Ivan Porro, Livia Torterolo, Marco Fato, Paolo Romano, Ulrich Pfeffer

**Affiliations:** 1 University of Genoa, Department of Communication, Computer and System Sciences, Viale Causa 13, Genoa, 16145, Italy; 2 National Research Council, Institute of Electronics and Engineering of Information and Telecommunications, Torre di Francia, Via de Marini 6, 16149, Genoa, Italy; 3 National Cancer Research Institute, Bioinformatics group, Largo Rosanna Benzi 10,16132, Genoa, Italy; 4 National Cancer Research Institute, Functional Genomics, Largo Rosanna Benzi 10, 16132, Genoa, Italy

## Abstract

**Background:**

Complex microarray gene expression datasets can be used for many independent analyses and are particularly interesting for the validation of potential biomarkers and multi-gene classifiers. This article presents a novel method to perform correlations between microarray gene expression data and clinico-pathological data through a combination of available and newly developed processing tools.

**Results:**

We developed Survival Online (available at ), a Web-based system that allows for the analysis of Affymetrix GeneChip microarrays by using a parallel version of dChip. The user is first enabled to select pre-loaded datasets or single samples thereof, as well as single genes or lists of genes. Expression values of selected genes are then correlated with sample annotation data by uni- or multi-variate Cox regression and survival analyses. The system was tested using publicly available breast cancer datasets and GO (Gene Ontology) derived gene lists or single genes for survival analyses.

**Conclusion:**

The system can be used by bio-medical researchers without specific computation skills to validate potential biomarkers or multi-gene classifiers. The design of the service, the parallelization of pre-processing tasks and the implementation on an HPC (High Performance Computing) environment make this system a useful tool for validation on several independent datasets.

## Background

High throughput gene expression analyses carried out by using innovative microarrays are increasingly used for the identification of biomarkers and for their combination in prognostic or predictive classifiers, so called signatures [1]. The usual design procedure of such studies consists in the following three steps: i) feature selection by statistical or artificial intelligence approaches, ii) development of the classifier on a training set, and iii) validation of the signature [2]. Suitable data sets are more and more available in microarray data repositories, mainly GEO (Gene Expression Omnibus) [3,4] and ArrayExpress [5,6].

These data sets can be used to study the correlation of clinico-pathological and follow-up data with gene expression both for the definition of signatures and to test hypotheses on the effects of over/under expression of single genes. This is particularly important in the light of the vast literature on potential biomarkers that are often tested on very limited patient cohorts and are not validated on independent data sets.

Different tools, implementing distinct algorithms, are available for diverse platforms.

Web-based software would avoid this problem, but up to date, apart from some exceptions (e.g. Expression Profiler [7]), most of web tools are spread over different servers and employ different file formats.

GEPAS [8] (Gene Expression Profile Analysis Suite) is an integrated web-based pipeline for the analysis of gene expression patterns aimed at putting the most popular tools together through an integrated interface. This allows end users to make transparently profit from the various tools, once their data has been uploaded. This solution, then, provides a powerful set of statistical services for gene expression analysis to users. However, the single analysis, for example survival analysis, presents some limitations concerning, e.g., availability of various tools' options, flexibility in the analysis flow and support for large datasets. Moreover, in order to customize their analyses, users should design ad-hoc pipelines, by making use of workflow management features, and this would imply more complex requirements and constraints.

Another tool, Asterias [9], provides a similar suite of applications, even if it has different implementation language and application interface; its emphasis is put on parallel computing.

We therefore set out to develop a flexible tool that allows to investigate large microarray datasets – or subsets thereof – by using standardized statistical approaches aimed at finding out the correlation of clinical data with gene expression results. In the final setting, researchers should be able to select either single or several datasets, or even single samples from different datasets, to create their own unique dataset. They would then be able to determine, on the basis of this new dataset, correlations between the expression of single genes or gene lists and clinical data.

To this aim, those limitations that are posed by current tools must also be taken into account. In fact, most of current statistical analyses on gene expression data is performed by using the R language, with the support of domain specific extension packages, such as Bioconductor [10] for life sciences. It is known that R poses serious limitations in dealing with large amount of data; for example, it indicates a memory failure when the required amount of memory exceeds the address-space limit for the process or, more likely, because the system is unable to provide a large enough contiguous block, even if there is enough free memory available. So, we designed a structured and engineered procedure that fully exploits available hardware and software resources thus avoiding many of the problems that are faced by researchers.

This limitation can be particularly relevant for normalization of microarray data and calculation of gene expression. We therefore developed a parallel version of dChip [11] for Linux that is able to handle datasets of the order of thousands of microarrays [12].

Microarray data analyses procedures are usually composed of several steps ranging from pre-processing (background correction, normalization, summarization) to the statistical analysis of differential gene expression. This may involve different computer programs, ranging from specialized tools and statistical software environments to simple spreadsheets. It is therefore difficult to embed them all into a single program or script. Even when possible, this requires time and skills, it is hard to maintain as well as unsuited for the dissemination to the scientific community. One of the most common solutions proposed to solve this issue is based on computerized scientific workflows. In this approach, users can design their virtual experiments using a specialized software that is able to make access to standardized services and to properly visualize resulting data. Web based solutions able to guarantee versioning and reproducibility of experiments have also been presented in order to build coherent collections of experiments to be used and re-used by the community. However, this approach implies that all services needed must already be available and provided either by the developer or by a third party. Additional concerns derive from overall maximum execution time and the amount of data that can be handled by services, determined by the protocols used for inter-processes messaging (SOAP, Simple Object Access Protocol) and for data transfer (HTTP, HyperText Transfer Protocol). FTP (File Transfer Protocol) and HTTP protocols, that are presently widely used on the Internet, were not designed for the transfer of large datasets. If used for large data set, transfer is slow due to the limits inherent in their processing overheads and the transport layer protocol, TCP (Transmission Control Protocol). This also poses limitations to tools that rely on such data transfer solutions (e.g., the Taverna Workbench [13]).

The goal of this research is to develop, set-up and validate a service for automating functional genomics analyses used in breast cancer survival studies and prognosis assessment. We present the strategy used for organizing heterogeneous computational steps by taking benefit from High Performance Computing (HPC) hardware and offering a simple user interface through a Web portal. The current implementation only exploits local resources; further work is underway to integrate the service within existing e-Science Grids, such as Enabling Grids for E-sciencE (EGEE) [14].

## Results

### The service

In this work, we present a methodology and an example of application successfully used to translate an existing procedure into an automated and consolidated Web based process which can benefit from existing High Performance Computing resources.

For the validation of our methodology, we explored the possibility of obtaining information on the correlation of single genes or lists of genes with disease free survival after surgical removal of primary breast cancers, as described in the Methods section, and implemented a web tool that we called Survival Online, The main processing steps, and other intermediate manual data preparation steps, that are necessary for such analyses, were analyzed, converted into web services and made available through the BMportal, a portal developed at the Biolab of the Department of Communication, Computer and System Sciences (DIST) of the University of Genoa, by using the EnginFrame framework [15]. This tool was chosen because it is the platform adopted by the Laboratory of Interdisciplinary Technologies for Bioinformatics (LITBIO) project [16] and, as a consequence, all newly developed services can be deployed without any additional effort in the LITBIO portal [17], thus reaching a large user community.

**Figure 1:**
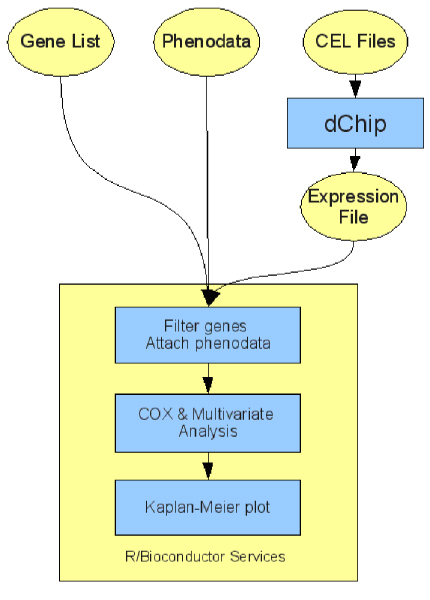
**Workflow of the analysis**. The sequence of the analysis steps performed is shown.

The automated procedure is shown in Figure 1 that includes the diagram of the sequence of the processing steps involved and their relationship. In the first step, the parallel version of dChip is used to pre-process microarray data (background correction and normalization) and to determine gene expression (by using the Perfect Match only algorithm). The following steps further evaluate expression values by means of several R/Bioconductor methods. First, under/over-expressed genes are filtered so that only a user defined set of genes is conserved. Then, specific phenodata are associated to samples. Finally, a Cox regression analysis, possibly combined with a multivariate analysis, is performed and the Kaplan-Meier survival plot is computed.

**Figure 2:**
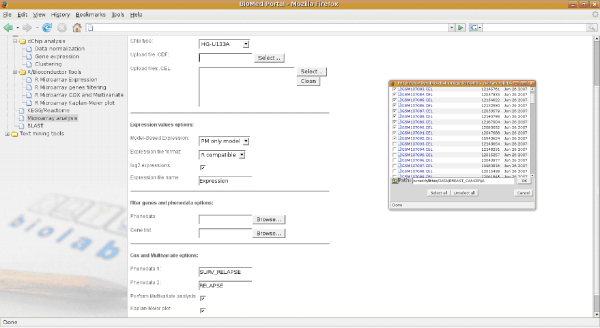
**Web portal screenshot 1**. The user interface for entering input parameters and selecting analysis options. Users can browse and select data remotely or upload it from their machine.

**Figure 3:**
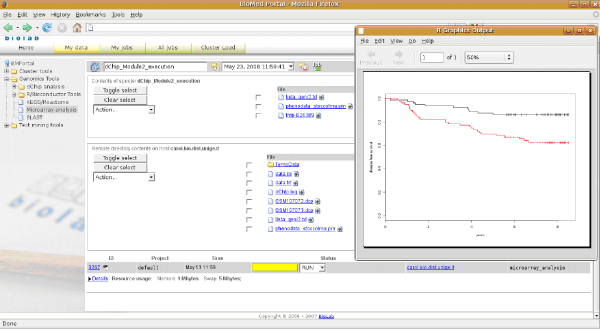
**Web portal screenshot 2**. The execution spooler of the portal. Both result files and execution status are visible.

In Figure 2, the user interface for entering input parameters and selecting analysis options is shown. Users can browse and select data remotely or upload it from their machine. In Figure 3, the execution spooler of the portal is shown. Both result files and execution status are visible.

### Testing

The automated procedure has been tested in order to assess its added value. To this end, a series of gene lists that were recently identified [18] was used. Multi-gene analyses, as those performed here, are the first step in signature development and need to be validated on independent data sets: this extended validation was not performed in this case. Results achieved by using Survival Online were identical to those obtained on local resources, thus indicating the correct functionality of the service.

Results were classified according to appropriate indicators. Increase of performance compared to the classic desktop-based processing was assessed by using quantitative indicators, such as time spent for analysis against different sizes of dataset, that showed a clear advantage in using Survival Online. Tables 1 and 2 shows the execution times obtained running the test analysis with both the traditional procedure and Survival Online (see Table 1). Table 2 presents a comparison between different analysis tools such as dChip, RMA (Robust Multi-array Average) and GCRMA (GC Robust Multi-array Average.

**Table 1:** Analysis execution times (using dChip for expression values computation)

**Indicator**	**Desktop PC**	**Web based service**	**Web based service (parallel dChip)**
50 microarrays	24 min	10 min	5 min
150 microarrays	46 min	29 min	9 min
400 microarrays	85 min	68 min	22 min

This table shows execution times related to the execution of the automated procedure by using a desktop PC, the web based service, the web based service with the parallel dChip using five parallel jobs. Execution on the desktop is carried on by an experienced operator on a system with all needed software already up and running. It includes intermediate manual operations and access to data stored locally on the PC.

Web execution times are calculated starting with the same operator already logged in to the service. Analysis is carried out on the same data, already available on the portal.

**Table 2:** Analysis tools comparison

**Indicator**	**dChip**	**RMA**	**GCRMA**
50 microarrays	√	√	X
150 microarrays	√	√	X
400 microarrays	√	X	X

This table shows the skill of dChip, RMA and GCRMA to execute the analysis based on increasing number of microarrays. GCRMA doesn't even allow to analyze 50 microarrays, while dChip succeeds in analyzing more than 400 microarrays.

Increase in usability, that is a key point in this work, since the designed service is intended to be used as an actual and effective support to translational research, was also assessed. Usability tests have been performed by staff of the Functional Genomics laboratory of the National Cancer Research Institute of Genoa, whose domain expertise afforded a real evaluation of the service. As shown in Table 3, a qualitative assessment of usability of the traditional manual procedure and of Survival Online was carried out. Users involved in the testing phase were asked to choose the level of satisfaction (poor, medium, good) with reference to three indicators: ease of use, repeatability and accessibility.

**Table 3:** Qualitative assessment of usability

***Indicator***	***Desktop PC***	***Web based service***
Ease of use	medium	good
Repeatability	poor	good
Accessibility	poor	good

Qualitative comparison between traditional approach and the equivalent Web based service is based on the opinions given by the users involved in the evaluation process. Evaluation scale is based on three levels: poor, medium, good. The comparison shows a clearly better usability of the Survival Online tool.

## Discussion

Expression analyses using complex oligonucleotide microarrays has profoundly changed biomedical research. Classical single gene studies necessarily start with a hypothesis, most often based on pre-existing or preliminary evidence or just on an intuition. Instead, high throughput approaches, such as microarray gene expression analyses, yield data sets that are usually created and queried for a specific scope, yet the data is available for other, completely unrelated analyses. Single gene studies for the identification of biomarkers for disease prognostication or prediction of treatment response have therefore led to an overwhelming number of publications where validation is usually limited to a single study on a single dataset [19].

However, prioritization of the many biomarkers for in depth validation and possible developments towards clinical application is difficult. This difficulty can be overcome by using publicly available microarray datasets with relevant clinical-pathological data that allow for in-silico validation of potential biomarkers for the selection of *bona fide *candidates for further development. Similarly, the same datasets can be used to analyze the classification power of multigene classifiers (signatures).

We presented here a web based system, Survival Online, where such analyses can easily be carried out by bio-medical researchers without special computation skills. At present, the system allows to select a whole dataset or a subset of it for a straightforward analysis of correlations between clinical and gene expression data. Classical statistical methods (uni- and multi-variate Cox-regression analysis, survival analysis, Kaplan-Meier curves) are combined in a pipeline to automate the manual procedure daily performed by researchers. The analytical tool box will be further enriched through the addition of advanced feature selection tools and of classification methods based on artificial intelligence approaches.

The validation of a classifier is better achieved when more independent data sets are taken into account. For this reason, our service is designed to harbour many different datasets to be used separately or for the generation of specific, user defined data (sub-)sets. At present, this is limited to the widely used Affymetrix GeneChip arrays. Other platforms can also be integrated, but under the condition that probesets can be matched between the different arrays by using specific conversion tools and by correcting for batch effects.

Analyses that use very large datasets or several smaller datasets together are normally limited by the computation power available. In our case, this limitation is overcome by the implementation of the service on a high performance platform and by the parallelization of the microarray data analysis suite dChip.

We have tested our system using a breast cancer dataset (see Methods section) retrieved from GEO. We were able to monitor the classification power of both gene sets selected from Gene Ontology categories and single genes selected from literature [18]. The data shows that the systems allows for straightforward testing of potential biomarkers.

Functional analyses of single genes or gene lists should always include an assessment of their effect on clinical outcome of cancer patients. Figures 2 and 3 illustrate that Survival Online enables the non expert biomedical researchers to perform such analyses using existing data sets.

In some cases, the proposed approach could be considered better than a classical workflow approach. First, the execution of the analysis is not demanded to a single execution engine on the computer of the user (e.g. Taverna client) or on a server (e.g. Taverna remote service, Moteur [20]). Approaches to execute workflows on distributed environments as a set of services exist (e.g. DAG jobs [21], P-grade portal [22]) but they are not implemented in HPC environments and are often affected by middleware overhead and network constraints. Since this case study requires a high level of interaction and usability, the best solution revealed to be the submission of a collection of single processes on HPC infrastructure synchronized by a scheduling system manager.

Results show that our approach can scale up in the size of processed data well above current available desktop computer limitations (up to 2 computing cores and 1 GB of RAM) just by exploiting a cluster made up of three nodes, each composed by a low end server (2 computing cores and 1 GB of RAM). Obviously, by using more resources it is either possible to achieve better results or, in some cases, to overcome the single machine limitations. However, since current trends in information technology (IT) are to offer software as a service and to concentrate computing power into data centers, due to cooling, power and skills requirements, it is essential to adequate existing procedures and applications accordingly. This will also avoid the spread of investments in computing equipment inside non-IT departments and will help to better exploit resources due to their concentration.

## Conclusion

Survival Online, allows for a straightforward execution of complex data correlations aimed at the identification of biomarkers and multi-gene classifiers. The system enables bio-medical researchers to perform validations on several independent microarray gene expression datasets in a simple and reliable fashion by using robust classical statistics. The collaboration between molecular oncologists and software engineers allowed for the optimisation of the system without loosing flexibility. The system relies on widely used analytical tools. Results obtained with the Web-based approach are identical to those achieved by using the traditional methods.

Future developments are planned to enhance service usability by using a standard WSDL (Web Services Description Language) interface. In fact, the adopted framework is able to provide services both through a Web interface and through a Web Service, exploitable from any Web Services enabled client application.

Orchestrating multiple processing steps in a workflow strategy opens a new scenario where users can design complex experiments, tailored to their needs, just by using portal services as workflow building bocks.

This is possible, e.g., by importing WSDL descriptions of services into a client application, such as the Taverna Workbench, and by letting the portal to execute and monitor the workflow life cycle, thus avoiding client side enacting limitations.

In the future, we plan to implement additional feature selection and classification tools and to add further datasets. The development of inter-array comparison tools is also planned.

## Methods

### Microarrays analysis

Microarray expression analyses of human breast cancers were performed on the dataset GSE1456 obtained from Gene Expression Omnibus [3]. The data set consists of 159 breast cancers from patients who received surgery at Karolinska Hospital from 1994 to 1996 [23]. Expression sets were calculated from raw microarray data, previously background corrected, normalized using invariant-set method and summarized using Perfect Match (PM) only algorithm implemented in dChip. Samples were annotated with patients' information, including data on disease relapse and overall and disease free survival, representing their "phenodata".

### Survival analysis

Expression levels of previously selected genes were combined in a weighted linear score, where weights were determined by the coefficients of a multivariate Cox regression model. The median value of the score was chosen as the cut off to classify patients at low or high risk of disease relapse. All analyses were performed with R/Bioconductor package Survival.

### Service validation

Results were classified according both to the test cases provided and to appropriate indicators. These included: (i) performance increase compared to the classic desktop-based processing (quantitative indicators, such as time spent for analysis against different sizes of dataset, have been adopted), (ii) increase of usability (qualitative-quantitative indicators were used, with reference both to reliability and to repeatability of experiments).

**Figure 4:**
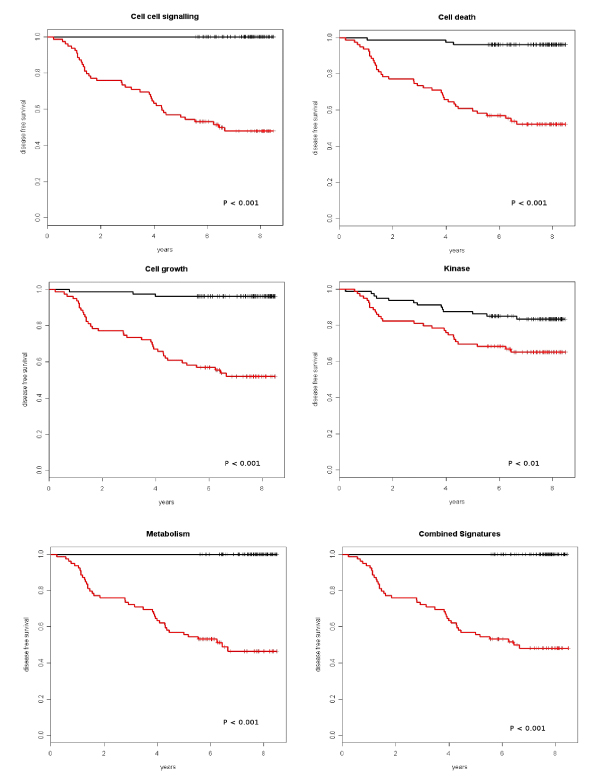
**Survival analyses using gene lists**. Gene lists were obtained from Gene Ontology categories as indicated. The "combined signature" contains the five most strongly relapse associated genes of the five other lists. Samples were divided into two classes based on the median values of the combined score of the multivariate model (black: good prognosis, red: poor prognosis).

Genes contained in the lists used for tests were selected by imposing both annotation to specified Gene Ontology categories (cell death, metabolism, kinase, cell cell signalling, cell growth) and significant association with the parameter "relapse" (p < 0.001) in the Stockholm dataset. The gene list "combined signatures" contains the five most strongly relapse associated genes from the above lists (for details see [18]) (Figure 4).

**Figure 5:**
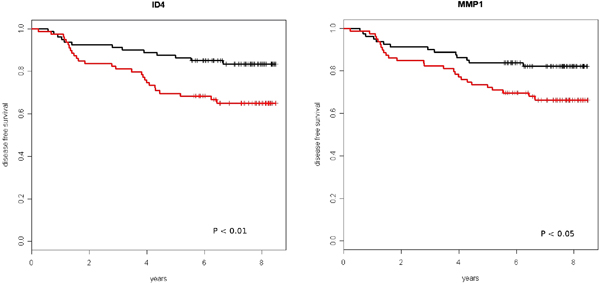
**Survival analysis using a single gene**. Expression values of the transcriptional regulator ID4 and of the matrix metalloprotease MMP1 were correlated with disease free survival. Samples were divided into two classes based on the median values respectively of ID4 and MMP1 expression (black: expression below the median value, red: above the median value).

For single gene analyses, we selected the transcriptional regulator ID4 that has been shown to regulate the expression of the tumour suppressor gene BRCA1 [24] (Figure 5).

### Development

Starting from user requirements and available components, it has been decided to build the service using BMportal, the biomedical portal framework available at Biolab [25,26], that offers basic services in different biomedical fields that can be combined into larger services covering complex procedures, as for the use case here presented.

BMportal includes sophisticated data management for all stages of job lifetime and is integrated with all relevant Grid and HPC workload management systems, using plug-in modules that extend its functionality leveraging the underlying infrastructure.

It provides a flexible presentation engine, solidly based on leading Web and XML (Extensible Markup Language) standards. This module provides access to the underlying services via multiple standard languages layered on HTTP, including HTML (HyperText Markup Language), SOAP and RSS (Really Simple Syndication).

The authorisation system is a core component of the architecture. It provides fine grained access control to all services via a role-based engine, implementing both presentation, execution and output processing layers.

The authentication system provides flexible delegation to a rich set of external authorities, and provides internal, session-level single-sign-on across multiple services.

The framework flexibility allows the integration of different tools and resources that can be orchestrated in customized complex experiments and provided as user-friendly web-based services.

Concerning this case study, LSF from Platform Computing [27] has been used as HPC scheduler for job submission and synchronization. Using the LSF plugin for EnginFrame, both R/Bioconductor and dChip tools can be submitted as LSF jobs to the HPC infrastructure, so that every step of the analysis can be easily monitored through the portal.

## Competing interests

The authors declare that they have no competing interests.

## Authors' contributions

IP, PR and UP contributed to the design of the service. LC and LT contributed to the development and testing of the service. UP and VM defined the clinical use case and its test and validation. MF coordinated the work at University of Genoa and UP did the same at the National Cancer Research Institute. LT drafted the paper, and UP, VM, PR revised it. All authors read and approved the final manuscript. All authors contributed equally to this paper.

## References

[B1] Ross JS, Hatzis C, Symmans WF, Pusztai L, Hortobagyi GN (2008). Commercialized multigene predictors of clinical outcome for breast cancer. Oncologist.

[B2] Dupuy A, Simon RM (2007). Critical review of published microarray studies for cancer outcome and guidelines on statistical analysis and reporting. J Natl Cancer Inst.

[B3] Gene Expression Omnibus (GEO). http://www.ncbi.nlm.nih.gov/geo/.

[B4] Edgar R, Domrachev M, Lash AE (2002). Gene Expression Omnibus: NCBI gene expression and hybridization array data repository. Nucleic Acids Res.

[B5] ArrayExpress. http://www.ebi.ac.uk/microarray-as/ae/.

[B6] Parkinson H, Kapushesky M, Shojatalab M, Abeygunawardena N, Coulson R, Farne A, Holloway E, Kolesnykov N, Lilja P, Lukk M, Mani R, Rayner T, Sharma A, William E, Sarkans U, Brazma A (2007). ArrayExpress – a public database of microarray experiments and gene expression profiles. Nucleic Acids Res.

[B7] Kapushesky M, Kemmeren P, Culhane AC, Durinck S, Ihmels J, Körner C, Kull M, Torrente A, Sarkans U, Vilo J, Brazma A (2004). Expression Profiler: next generation – an online platform for analysis of microarray data. Nucleic Acids Res.

[B8] Tárraga J, Medina I, Carbonell J, Huerta-Cepas J, Minguez P, Alloza E, Al-Shahrour F, Vegas-Azcárate S, Goetz S, Escobar P, Garcia-Garcia F, Conesa A, Montaner D, Dopazo J (2008). GEPAS, a web-based tool for microarray data analysis and interpretation. Nucleic Acids Res.

[B9] Díaz-Uriarte R, Alibés A, Morrissey ER, Cañada A, Rueda OM, Neves ML (2007). Asterias: integrated analysis of expression and aCGH data using an open-source, web-based, parallelized software suite. Nucleic Acids Res.

[B10] Gentleman RC, Carey VJ, Bates DM, Bolstad B, Dettling M, Dudoit S, Ellis B, Gautier L, Ge Y, Gentry J, Hornik K, Hothorn T, Huber W, Iacus S, Irizarry R, Leisch F, Li C, Maechler M, Rossini AJ, Sawitzki G, Smith C, Smyth G, Tierney L, Yang JY, Zhang J (2004). Bioconductor: open software development for computational biology and bioinformatics. Genome Biol.

[B11] Li C, Wong WH, Parmigiani G, Garrett ES, Irizarry R, Zeger SL (2003). DNA-Chip Analyzer (dChip). The analysis of gene expression data: methods and software.

[B12] Corradi L, Fato M, Porro I, Scaglione S, Torterolo L (2008). A Web-based and Grid-enabled dChip version for the analysis of large sets of gene expression data. BMC Bioinformatics.

[B13] Oinn TM, Addis M, Ferris J, Marvin D, Senger M, Greenwood RM, Carver T, Glover K, Pocock MR, Wipat A, Li P (2004). Taverna: a tool for the composition and enactment of bioinformatics workflows. Bioinformatics.

[B14] Enabling Grids for E-sciencE (EGEE) project. http://www.eu-egee.org/.

[B15] Melato M, Glatard T, Falzone A, Torterolo L Genius Portal and EnginFrame Framework: new features and future perspectives. Proceedings of EGEE06, International Conference Centre Geneva (CICG) – Geneva, Switzerland, September 2006.

[B16] LITBIO – Laboratory for Interdisciplinary Technologies in Bioinformatics FIRB Project. http://www.litbio.org.

[B17] LITBIO Portal. http://portal.litbio.org/.

[B18] Pfeffer U, Romeo F, Noonan DM, Albini A (2009). Prediction of breast cancer metastasis by genomic profiling: where do we stand?. Clin Exp Metastasis.

[B19] Sutcliffe P, Hummel S, Simpson E, Young T, Rees A, Wilkinson A, Hamdy F, Clarke N, Staffurth J (2009). Use of classical and novel biomarkers as prognostic risk factors for localised prostate cancer: a systematic review. Health Technology Assessment.

[B20] Glatard T, Montagnat J, Pennec X (2006). Medical image registration algorithms assesment: Bronze Standard application enactment on grids using the MOTEUR workflow engine. Stud Health Technol Inform.

[B21] Hantz F Light P2P platform of computing for DAG. Distributed Frameworks for Multimedia Applications, May 2006 The 2nd International Conference on, 2006.

[B22] P-Grade Portal. http://portal.p-grade.hu/.

[B23] Pawitan Y, Bjohle J, Amler L, Borg AL, Egyhazi S, Hall P, Han X, Holmberg L, Huang F, Klaar S, Liu ET, Miller L, Nordgren H, Ploner A, Sandelin K, Shaw PM, Smeds J, Skoog L, Wedren S, Bergh J (2005). Gene expression profiling spares early breast cancer patients from adjuvant therapy: derived and validated in two population-based cohorts. Breast Cancer Res.

[B24] Beger C, Pierce LN, Kruger M, Marcusson EG, Robbins JM, Welcsh P, Welch PJ, Welte K, King MC, Barber JR, Wong-Staal F (2001). Identification of Id4 as a regulator of BRCA1 expression by using a ribozyme-library-based inverse genomics approach. Proc Natl Acad Sci USA.

[B25] Porro I, Torterolo L, Corradi L, Fato M, Papadimitropoulos A, Scaglione S, Schenone A, Viti F (2007). A Grid-based solution for management and analysis of microarrays in distributed experiments. BMC Bioinformatics.

[B26] Beltrame F, Papadimitropoulos A, Porro I, Scaglione S, Schenone A, Torterolo L, Viti F (2007). GEMMA – A Grid environment for microarray management and analysis in bone marrow stem cells experiments. Future Generation Computer Systems.

[B27] Platform LSF. http://www.platform.com/.

